# Dual targeting of PI3K and MEK enhances the radiation response of *K-RAS* mutated non-small cell lung cancer

**DOI:** 10.18632/oncotarget.9670

**Published:** 2016-05-27

**Authors:** Mahmoud Toulany, Mari Iida, Simone Keinath, Firdevs F. Iyi, Katharina Mueck, Birgit Fehrenbacher, Wael Y. Mansour, Martin Schaller, Deric L. Wheeler, H. Peter Rodemann

**Affiliations:** ^1^ Division of Radiobiology and Molecular Environmental Research, Department of Radiation Oncology, University of Tuebingen, Tuebingen, Germany; ^2^ Department of Human Oncology, University of Wisconsin School of Medicine and Public Health, Wisconsin Institute for Medical Research, Madison, WI, USA; ^3^ Department of Dermatology, University of Tuebingen, Tuebingen, Germany; ^4^ Tumor Biology Department, National Cancer Institute, Cairo University, Cairo, Egypt; ^5^ Laboratory of Radiobiology and Experimental Radiooncology, University Medical Center Hamburg-Eppendorf, Hamburg, Germany

**Keywords:** NSCLC, PI3K/Akt, MAPK/ERK, radiotherapy, double-strand breaks

## Abstract

Despite the significant contribution of radiotherapy to non-small lung cancer (NSCLC), radioresistance still occurs. One of the major radioresistance mechanisms is the hyperactivation of the PI3K/Akt pathway in which Akt facilitates the repair of DNA double-strand breaks (DSBs) through the stimulation of DNA-PKcs. We investigated if targeting PI3K would be a potential approach for enhancing the radiosensitivity of *K-RAS* mutated (K-RASmut) NSCLC cell lines A549 and H460.

Short-term (1-2 h) pre-treatment of cells with the PI3K inhibitor PI-103 (1 μM) inhibited Akt/DNA-PKcs activity, blocked DSBs repair and induced radiosensitivity, while long-term (24 h) pre-treatment did not. Lack of an effect after 24 h of PI-103 pre-treatment was due to reactivation of K-Ras/MEK/ERK-dependent Akt. However, long-term treatment with the combination of PI-103 and MEK inhibitor PD98059 completely blocked reactivation of Akt and impaired DSBs repair through non-homologous end joining (NHEJ) leading to radiosensitization. The effect of PI3K inhibition on Akt signaling was also tested in A549 mouse xenografts. P-Akt and P-DNA-PKcs were inhibited 30 min post-irradiation in xenografts, which were pretreated by PI-103 30 min before irradiation. However, Akt was reactivated 30 min post-irradiation in tumors, which were pre-treated for 3 h with PI-103 before irradiation. After a 24 h pretreatment with PI-103, a significant reactivation of Akt was achieved 24 h after irradiation. Thus, due to MEK/ERK-dependent reactivation of Akt, targeting PI3K alone is not a suitable approach for radiosensitizing K-RASmut NSCLC cells, indicating that dual targeting of PI3K and MEK is an efficient approach to improve radiotherapy outcome.

## INTRODUCTION

The *RAS* isoforms (*K-RAS, H-RAS* and *N-RAS*) have point mutations in nearly 30% of all human tumors and are small molecular weight GTPases that couple extracellular signals to intracellular effector pathways [1]. It is known that *K-RAS* mutation leads to constitutive K-Ras activity and is associated with stimulated autocrine production of the epidermal growth factor receptor (EGFR) ligand, amphiregulin [2]. The phosphatidylinositol 3-kinase (PI3K)/Akt and MEK/ERK pathways are the major effectors of oncogenic RAS involved in tumor cell clonogenic activity. Constitutive activation of these pathways leads to resistance to EGFR molecular targeting strategies such as the anti-EGFR antibody cetuximab or the EGFR-tyrosine kinase (EGFR-TK) inhibitors gefitinib and erlotinib [3]. Preclinical studies demonstrate that constitutive K-Ras activity leads to accelerated repair of DNA double-strand breaks (DSBs) [4] and increased radiotherapy resistance [5–7]. Clinical data indicates that most of cancer patients with *K-RAS* mutations have significantly worse recurrence-free survival and distant metastases following radiotherapy [8]. In a previous report, we demonstrated that, like the production of the EGFR ligand amphiregulin in *K-RAS* mutated (K-RASmut) tumor cells, [2, 9] stimulated amphiregulin secretion is also obvious in head and neck squamous (HNSCC) tumor cells overexpressing *K-RAS* wild-type (K-RASwt). Thus, K-Ras hyperactivity induces Akt activation either through an EGFR/PI3K-dependent pathway [2, 10] or through H-RAS-dependent direct activation of the PI3K pathway [11, 12].

Due to crosstalk between the PI3K/Akt and MEK/ERK pathways, the inhibition of one pathway can lead to negative feedback and reactivation of another pathway. For example, MEK signaling can restore the expression of the phosphatase and tensin homolog (PTEN), both *in vitro* and *in vivo* [13]. Thus, recruitment of PTEN to the cell membrane is reduced because of MEK inhibition, which results in increased PI3K accumulation and Akt activation [13, 14]. It is known that combined MEK and Akt inhibition improves antitumor efficacy *in vivo* [15]. We previously reported that long-term inhibition of PI3K leads to EGFR/PI3K-independent but MEK/ERK-dependent reactivation of Akt in tumor cells presenting constitutive K-Ras activity either through *K-RAS* mutation or overexpression of K-RASwt [16]. Reactivation of the downstream components of PI3K such as Akt leads to limited effectiveness of the PI3K inhibitor PI-103 on the clonogenicity of tumor cells [16].

The effect of PI3K activity on DNA repair was suggested to occur through the stimulation of DNA-PK [17]. In this context, it could be demonstrated that activation of the PI3K/Akt pathway after exposure to ionizing radiation leads to efficient repair of DSBs through non-homologous end joining (NHEJ) as well as homologous recombination repair pathways [18, 19]. We first reported that activated Akt-mediated DNA damage repair can be suppressed by pharmacological or genetic targeting of Akt through the inhibition of DNA-PKcs [20–22]. Other reports also support our findings concerning the role of PI3K and/or Akt in DSBs repair and radioresistance [18, 19, 23–27].

Based on the reactivation of Akt following long-term (24 h) inhibition of PI3K [16], in the present study, we investigated whether PI3K targeting combined with irradiation remains an effective approach to impair the radioresistance of K-RASmut cells. Our *in vitro* and *in vivo* data indicated that short-term treatment with PI-103 inhibits Akt/DNA-PKcs activity after irradiation but long-term treatment does not. This leads to diminished DSBs repair and induces radiosensitivity after short-term inhibition of PI3K. Akt reactivation disturbs the targetability of PI3K and results in DSBs repair through NHEJ. MEK/ERK-dependent reactivation of Akt following long-term treatment led us to conclude that dual targeting of PI3K and MEK might be an effective approach to block NHEJ-dependent DSBs repair in NSCLC with a point mutation in the *K-RAS* gene.

## RESULTS

### Long-term inhibition of PI3K leads to PI3K-independent reactivation of Akt in K-RASmut NSCLC cell lines

**Figure 1:**
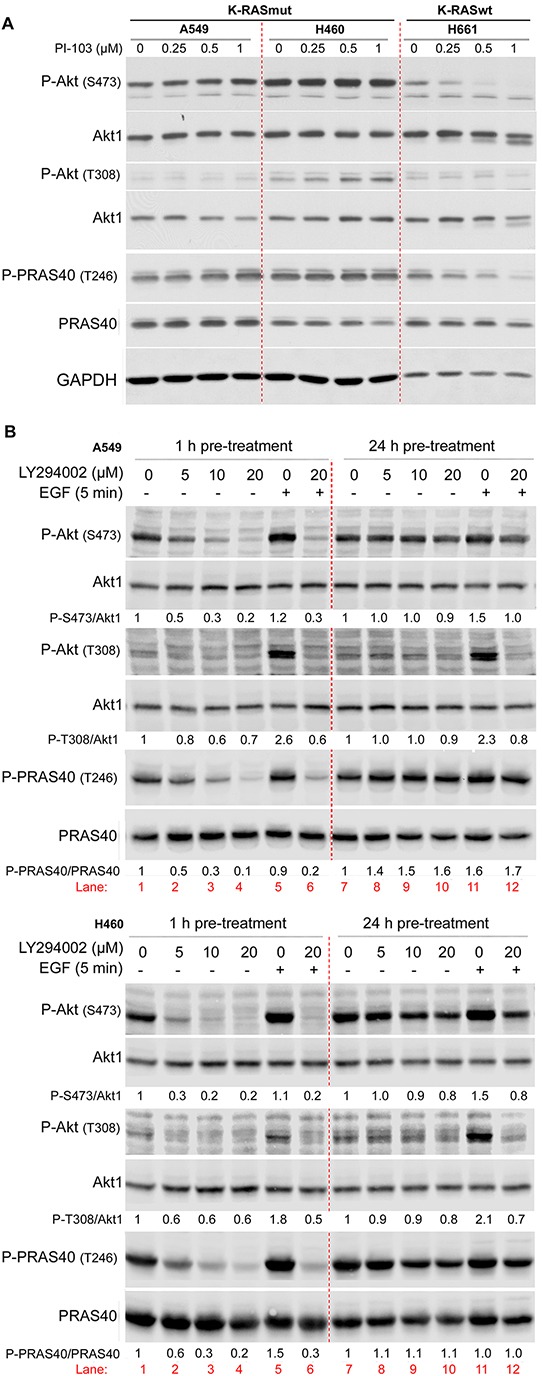
Long-term inhibition of PI3K leads to PI3K-independent reactivation of Akt NSCLC cell lines were treated with the indicated concentrations of PI-103 for 24 h **A.** and LY294002 for 2 h (lanes 1 to 4) or 24 h (lanes 7 to 10), **B.** and protein samples were isolated. In panel B, after treatment with LY294002 for 2 h (lane 6) or for 24 h (lane 12), cells were stimulated with EGF (100 ng/ml, 5 min) (lanes 5, 6, 11, 12), and protein samples were isolated. The levels of P-Akt (S473, T308) and P-PRAS40 (T246) were analyzed by Western blotting. The blots were stripped and incubated with antibodies against Akt1 and PRAS40, respectively. In panel B, P-PRAS40 and PRAS40 were detected in membranes from two SDS polyacrylamide gels loaded with the same protein sample. The densitometry values in panel B represent the ratio of phospho-S473/Akt1, phospho-T308/Akt1 or P-PRAS40/PRAS40 normalized to 1 in LY294002 non-treated controls. The concentration of DMSO as a vehicle in all treatment conditions was calculated to be 0.2%.

Previously, we demonstrated that long-term (24 h) treatment with the PI3K inhibitor PI-103 in tumor cells with constitutive K-Ras activity led to Akt reactivation [16]. In the present study, the effect of long-term inhibition of PI3K by PI-103 (24 h treatment) on the phosphorylation of Akt (S473 and T308) and its substrate PRAS40 at T246 was compared in K-RASmut NSCLC cell lines A549 and H460 with those in *K-RAS* wild-type (K-RASwt) H661 cells. Therefore, cells were treated with PI-103 (0.25, 0.5 and 1 μM) for 24 h. PI-103 did not inhibit the phosphorylation of Akt and PRAS40 in K-RASmt cells, while phosphorylation of both proteins in K-RASwt cells was inhibited in a dose-dependent manner (Figure 1A).

PI-103 may target mTOR in parallel to inhibiting PI3K. Because targeting mTOR complex 1 (mTORC1) by rapamycin can also lead to the reactivation of Akt [28, 29] it remains unclear whether Akt reactivity after treatment with PI-103 is due to the inhibition of PI3K or occurs due to the inhibition of mTORC1. To answer this question, the effect of LY294002 as a specific PI3K inhibitor on Akt phosphorylation was tested following 1 h and 24 h pre-treatment of A549 and H460 cells. As expected, a 1 h treatment with LY294002 inhibited the phosphorylation of Akt and PRAS40 in both cell lines in a dose-dependent manner (Figure 1B, lane 1 to lane 4). In contrast, treatment with LY294002 for 24 h did not inhibit the phosphorylation of Akt and PRAS40 (Figure 1B, lane 7 to lane 10). A 24 h pre-treatment with PI-103 may lead to the inactivation of the inhibitors and, consequently, a lack of effect on Akt activity rather than reactivation of Akt through a PI3K-independent pathway. To rule out this possibility, cells were treated with vehicle (Figure 1B, lane 5 and lane 11) or with LY294002 (20 μM) (Figure 1B, lanes 6 and 12) either for 1 h (lanes 5 & 6) or for 24 h (lanes 11 & 12), followed by stimulation with EGF for 5 min (lanes 5, 6, 11 and 12). These data indicate that EGF treatment in both cell lines led to phosphorylation of Akt (S473 and T308) (lanes 5 & 11) when compared to the non-stimulated condition in lane 1. Pre-treatment with LY294002 for 1 h inhibited basal phosphorylation as well as EGF-induced Akt phosphorylation (lane 6). Interestingly, as reflected by densitometry analysis, EGF-induced phosphorylation of Akt at S473 and T308 was completely blocked by LY294002 following 24 h pre-treatment (lane 12). This dataset indicates that PI3K activity remains blocked by PI3K inhibitors PI-103 or LY294002 during 24 h pre-treatment, and thus, reactivation of Akt is PI3K independent.

### Targeting of the components of the K-RAS/MEK/ERK pathway in irradiated cells blocks PI3K-independent reactivation of Akt

**Figure 2:**
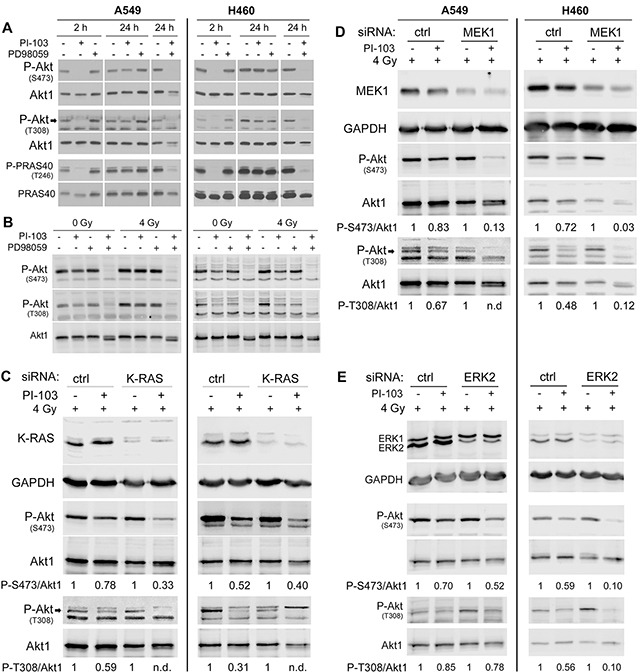
PI3K-independent reactivation of Akt is blocked by targeting of the components of K-RAS/MEK/ERK pathway in irradiated K-RASmut cells **A.** Cells were treated with PI-103 (PI) (1 μM), PD98059 (PD) (20 μM) and the combination of PI and PD for indicated time points and protein samples were isolated. **B.** Cells were treated with PI, PD or the combination of PI and PD for 24 h and were mock irradiated or irradiated with 4 Gy. Protein samples were isolated 10 min post-irradiation. **C-E.** Cells were transfected with 50 nM of non-targeting-siRNA or K-RAS-siRNA (C) non-targeting-siRNA or MEK1-siRNA (D) andnon-targeting-siRNA or ERK2-siRNA (E). Treatment with or without PI-103 (1 μM) was performed 48 h after transfection. Cells were irradiated with 4 Gy after treatment with PI-103 for 24 h. Protein samples were isolated 10 min post-irradiation. The level of P-Akt (S473), P-Akt (T308) and P-PRAS40 (T246) in the indicated experiments were analyzed by Western blotting. The blots were stripped and incubated with antibodies against Akt1 or PRAS40. In the siRNA experiments GAPDH was detected as a loading control. Densitometry values for phosphorylated Akt represent the ratio of P-Akt-S473/Akt1 and P-Akt-T308/Akt1 normalized to 1 in PI-103 non-treated controls.

Next, we examined if Akt reactivation in K-RASmut cells could be blocked by combinatorial treatment with PI3K inhibitor PI-103 and the MEK inhibitor PD98059. Akt activation was blocked by 2 h pre-treatment with PI-103 (PI), while 24 h pretreatment led to reactivation of Akt and the Akt substrate PRAS40 in A549 and H460 K-RASmut NSCLC cell lines. Treatment with PD98059 (PD) alone did not inhibit Akt and PRAS40 phosphorylation, while phosphorylation of both proteins was blocked when PD was combined with PI (Figure 2A).

It is well known that clinically relevant doses of ionizing radiation (IR) also induce activation of the PI3K/Akt pathway [2, 30] and Akt activity enhances post-irradiation cell survival. Therefore, we investigated whether MEK/ERK targeting abrogates Akt reactivation following 24 h pretreatment with PI-103 in irradiated NSCLC cells. To this end, cells were treated with PI-103 (1 μM), PD98059 (20 μM) or the combination of both inhibitors for 24 h, and mock irradiated or irradiated with 4 Gy. Level of P-Akt at S473 and T308 was tested 10 min after irradiation. In A549 cells, neither PI-103 nor PD98059 blocked Akt phosphorylation. However, combination of both inhibitors completely blocked Akt phosphorylation under both un-irradiated and irradiated conditions. In H460 cells, reactivation of Akt in irradiated cells was more apparent than in un-irradiated cells. However, combination of PI-103 and PD98059 completely blocked Akt phosphorylation in both un-irradiated and irradiated cells. Likewise, no inhibitory effect of PD98059 on Akt phosphorylation could be observed. In further experiments, the dependence of Akt reactivation after 24 h of PI3K inhibition of the K-Ras/MEK/ERK pathway in irradiated cells was investigated using siRNA. Knockdown of K-Ras (Figure 2C), MEK1 (Figure 2D) or ERK2 (Figure 2E) led to an efficient inhibition of Akt phosphorylation by PI-103 in combination with radiation exposure.

### Short-term treatment with PI3K inhibitor PI-103 inhibits radiation-induced DNA-PKcs activity

**Figure 3:**
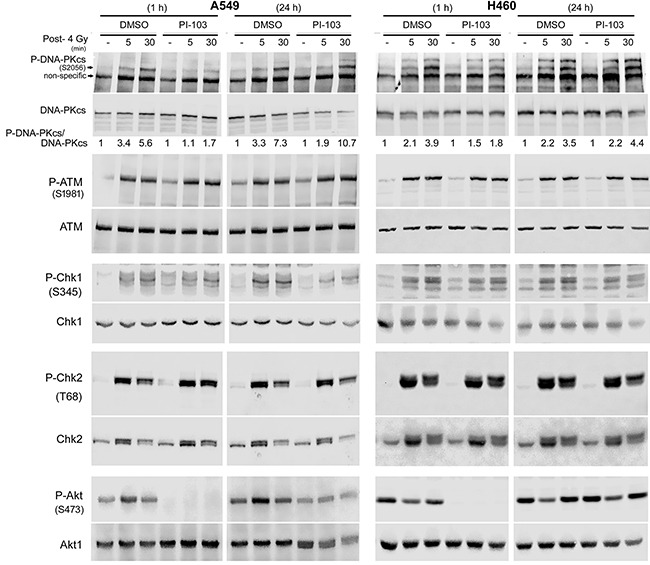
Long-term treatment with PI-103 leads to the lack of effect on Akt and DNA-PKcs phosphorylation after irradiation in K-RAS mutated cells Cells were treated with PI-103 for 1 h or 24 h and mock irradiated or irradiated with 4 Gy. Protein samples were isolated at the indicated time-points after irradiation and subjected to SDS-PAGE. The levels of P-DNA-PKcs (S2056), P-Akt (S473), P-ATM (S1981), P-Chk1 (S345) and P-Chk2 (T68) were analyzed by Western blotting. Blots were stripped and incubated with antibodies against DNA-PKcs, Akt1, ATM, Chk1 and Chk2. The densitometry values of P-DNA-PKcs/DNA-PKcs have been normalized to 1 in mock-irradiated conditions.

DNA-PKcs is a key enzyme involved in repair of DSBs after exposure to irradiation through NHEJ. It has been reported that activation of DNA-PKcs following irradiation leads to its autophosphorylation at S2056, which is partially dependent on Akt activity [22, 23]. Next, we tested whether short-term or long-term treatment with PI-103 differentially inhibits DNA-PKcs autophosphorylation. As shown in Figure 3, strong inhibition of P-DNA-PKcs was achieved by 1 h treatment with PI-103 in K-RASmut A549 and H460 cells. Figure 3 also indicates that diminished P-Akt levels after a 1 h pre-treatment with PI-103 could be associated with the inhibition of radiation-induced DNA-PKcs-S2056 phosphorylation at 5 min and 30 min post-irradiation in A549 and H460 cells. DNA-PKcs-S2056 phosphorylation did not change after irradiation following pretreatment with PI-103 for 24 h (Figure 3). Exposure to ionizing radiation induced the phosphorylation of ATM and the phosphorylation of the checkpoint proteins ChK1 and Chk2 in both cell lines, which were not inhibited after PI-103 treatment (Figure 3). Furthermore, we investigated whether differential effect of PI-103 on DNA-PKcs phosphorylation presented in Figure 3 is a K-RASmut specific event. To this end, K-RASwt H661 cells were treated with PI-103 for 2 h or for 24 h and irradiated with 4 Gy. Analyzing phosphorylation of DNA-PKcs at 5 min and 30 min post-irradiation revealed that PI-103 does not inhibited DNA-PKcs phosphorylation at S2056 (Supplementary Figure S1). As expected, PI-103 blocked phosphorylation of Akt after 2 h as well as after 24 h pretreatment conditions (Supplementary Figure S1).

### Short-term treatment with PI-103 inhibits Akt reactivation and DNA-PKcs phosphorylation *in vivo*

**Figure 4:**
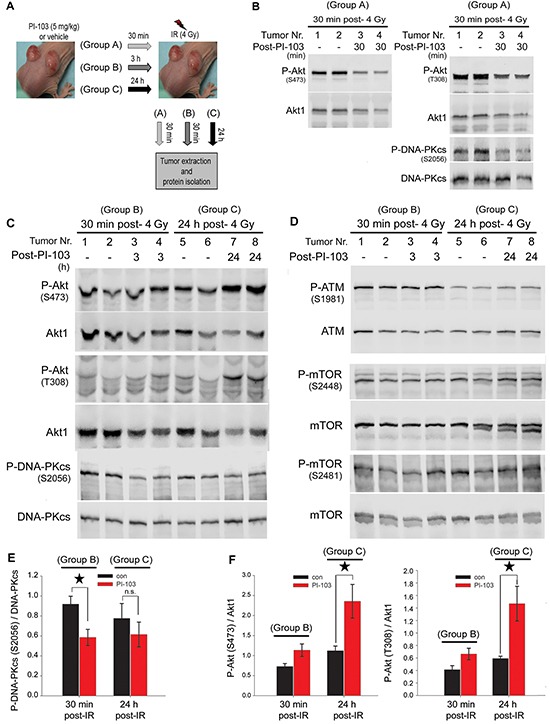
Long-term treatment with PI-103 leads to the reactivation of Akt and the lack of effect on radiation-induced DNA-PKcs phosphorylation *in vivo* **A.** Nude mice were injected with A549 (2 × 10^6^ cells) in both hind legs, and tumor diameters were measured weekly until they reached approximately 250 mm^3^. Thereafter, animals were treated with either vehicle or PI-103 (5 mg/kg) for 30 min, 3 h or 24 h, and tumors were locally irradiated with 4 Gy. At either 30 min or 24 h after irradiation, tumors were extracted, and protein samples were isolated. **B-D.** Protein expression levels of P-Akt, P-DNA-PKcs, P-mTOR and P-ATM were analyzed by Western blotting. Blots were stripped and incubated with the antibodies against DNA-PKcs, Akt1, ATM and mTOR, respectively. **E-F.** Histograms represent mean densitometry values of the ratio of P-DNA-PKcs (S2056) to total DNA-PKcs (E) and P-Akt (S473)/Akt1 as well as P-Akt (T308)/Akt1 (F) in irradiated tumors pre-treated with vehicle vs. irradiated tumors pre-treated with 5 mg/kg of PI-103, Error bars: SEM (*P<0.05, Student's *t*-test, n = 4 tumors).

To investigate the status of Akt reactivity following short-term or long-term inhibition of the PI3K pathway *in vivo*, an A549 mouse xenograft study was performed. Mice were injected with A549 cells as described in the Materials and Methods section. Tumors were allowed to grow to a volume of approx. 250 mm^3^ (Figure 4A). Thereafter, mice were gavaged with 5 mg/kg of PI-103 or vehicle, and after 30 min (group A), 3 h (group B) or 24 h (group C), tumors were locally irradiated with a single dose of 4 Gy. The tumors were extracted either 30 min post-irradiation (group A and group B) or 24 h post-irradiation (group C). Protein samples were isolated for Western blotting analysis. Immunoblot analysis indicated that the phosphorylation levels of Akt (S473 and T308) as well as phospho-DNA-PKcs (S2056) were reduced in tumors from group A (Figure 4B). In tumors from group B, the level of Akt phosphorylation at S473 (p = 0.058) and T308 sites (p = 0.067) was not significantly affected (Figure 4C & the densitometry data in Figure 4F), but PI-103 significantly decreased the level of P-DNA-PKcs (Figure 4C & the densitometry data in Figure 4E). In group C, long-term (24 h) pretreatment with PI-103 led to enhanced phosphorylation of Akt at both phosphorylation sites (p < 0.05) (Figure 4C & the densitometry data in Figure 4F) and a lack of inhibition of P-DNA-PKcs, analyzed at 24 h post-irradiation (p = 0.44) (Figure 4C & the densitometry in Figure 4E). Similar to the *in vitro* data (Figure 3), PI-103 did not inhibit ATM phosphorylation either at 30 min or at 24 h post-irradiation (Figure 4D). Likewise, PI-103 did not block the phosphorylation of mTOR at S2448 and S2481 in tumors from group A or from group C (Figure 4D).

### Short-term inhibition of Akt leads to the retention of phosphorylated DNA-PKcs at the DSB sites

**Figure 5:**
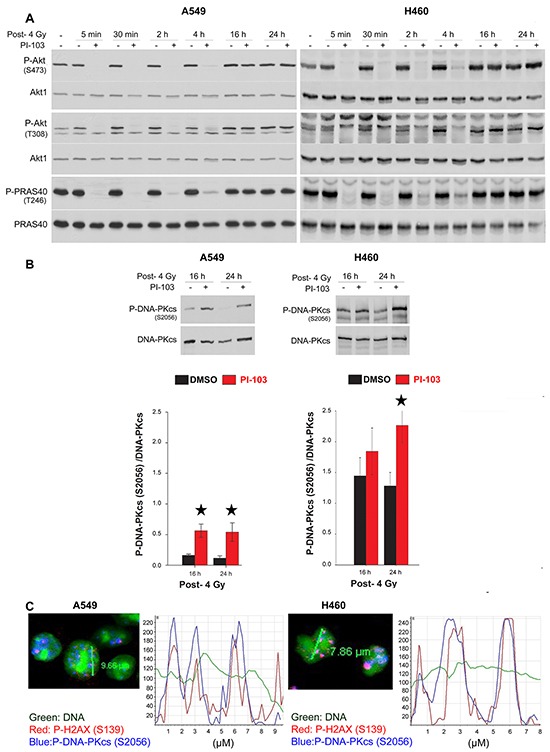
Short-term inhibition of Akt leads to the retention of phosphorylated DNA-PKcs to the DSBs site A. Cells were treated with DMSO and PI-103 for 2 h at different times and irradiated with 4 Gy. Protein samples were isolated from all treated cells at the same time at indicated time-points after irradiation with 4 Gy as well as from un-irradiated control and subjected to SDS-PAGE. The levels of P-Akt (S473), P-Akt (T308) and P-PRAS40 (T246) were analyzed by Western blotting. The blots were stripped and incubated with antibodies against corresponding total proteins. **B.** Cells were treated with DMSO and PI-103 for 2 h and irradiated with 4 Gy. At 16 and 24 h post-IR, levels of P-DNA-PKcs and DNA-PKcs were analyzed. The densitometry values represent the mean ratios of P-DNA-PKcs (S2056)/DNA-PKcs from 3 independent experiments. Error bars: SEM (*P <0.05, Student's *t*-test). **C.** A549 and H460 cells were treated with PI-103 for 2 h and irradiated with 4 Gy. Twenty-four hours after irradiation, immunofluorescence of P-DNA-PKcs (S2056) and γ-H2AX was performed. Representative confocal microscopy images and related intensity graphs indicate co-localization of P-DNA-PKcs with γ-H2AX foci.

Impairment of DNA-PKcs autophosphorylation results in DSBs repair deficiency and the lack of dissolved DNA repair proteins such as phosphorylated DNA-PKcs from DSB sites [31]. We previously reported an increased number of DNA-PKcs-S2056 foci co-localized with γ-H2AX in DSB sites after Akt1 knockdown [22]. Here, we tested whether the inhibition of DNA-PKcs phosphorylation following short-term treatment with PI-103 *in vitro* (Figure 3) and *in vivo* (Figure 4) led to persistent phosphorylation of DNA-PKcs 24 h post-irradiation. Time-kinetics analyses revealed that in cells that were pretreated for 2 h and irradiated, p-Akt was inhibited from 5 min to 4 h post-irradiation (Figure 5A). At 16 h and 24 h post-irradiation, reactivation of both Akt and PRAS40 was observed. Data presented in Figure 5B indicate that the inhibition of Akt by PI-103 at the time of irradiation blocks repair of DSBs leading to significant persistence of P-DNA-PKcs at DSB sites in both cell lines shown at 24 h post-irradiation (Figure 5B). A confocal microscopy study at 24 h post-irradiation confirmed the Western blot data and indicated that residual DNA-PKcs-S2056 foci are co-localized with γ-H2AX (S139) as a marker of DSBs after treatment with PI-103 for 2 h, followed by irradiation (Figure 5C).

### Long-term inhibition by the combination of PI-103 and PD98059 inhibits DSBs repair through NHEJ and induced radiosensitization in K-RASmut NSCLC cells

**Figure 6:**
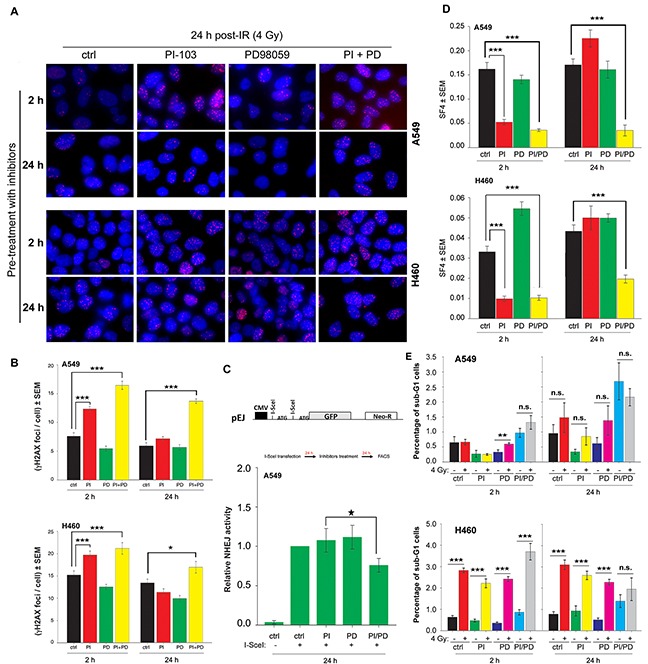
Long-term inhibition by the combination of PI-103 and PD98059 treatment inhibits DSBs repair through NHEJ and induced radiosensitization of K-RASmut NSCLC cells **A-E.** Cells were treated with PI-103 (1 μM), PD98059 (20 μM) and the combination of PI-103 and PD98059 for 2 h or 24 h. **A-B.** Cells were irradiated with 4 Gy and applied for analysis of residual DSBs using a γH2AX foci assay 24 h after irradiation. Images shown in Figure 6A are representative of residual γH2AX foci 24 h post-4 Gy in control cells or cells pretreated for 2 h or 24 h with the indicated inhibitors. The histograms shown in Figure 6B represent the mean number of foci per cells counted at least from 100 nuclei. **C.** Data represent the mean value of GFP expression from 6 independent experiments after 24 h treatment with DMSO, PI, PD or the combination of PI and PD in the NHEJ assay, as described in the *Materials and Methods* section. **D.** Survival fraction after 4 Gy (SF4) from clonogenic assay performed 2 h post-4 Gy. In A549 and H460 cells with 2 h pre-treatment as well as in A549 cells with 24 h pre-treatment, the SF4 value is the mean survival fraction obtained from 24 data points from two independent experiments (12 parallel data points from each experiment). In H460 cells treated for 24 h with the inhibitors, SF4 is the mean of 36 data points obtained from three independent experiments. **E.** Mean percentage of apoptosis from 9 data points obtained in 3 independent experiments, 48 h after mock irradiation or irradiation with 4 Gy in cells that were pretreated with DMSO, PI, PD or the combination of PI and PD for 2 h or 24 h.(*P <0.05, **P <0.01, ***P <0.001, Student's *t*-test); (ctrl: control, PI: PI-103, PD: PD98059); bars: SEM; n.s.: not significant.

Next, we investigated whether the persistence of DNA-PKcs phosphorylation at 24 h post-irradiation in cells pre-treated for 2 h with PI-103 (Figure 5) is associated with non-repaired DSBs. As shown by the γ-H2AX focus assay (Figure 6A-6B), pre-treatment with PI-103 for 2 h but not for 24 h strongly inhibited the repair of DSBs. To test whether the lack of effect of PI-103 after 24 h pre-treatment on DSBs repair is due to the MEK-dependent reactivation of Akt (Figure 2), NHEJ activity and residual γ-H2AX were analyzed 24 h after treatment with the MEK inhibitor PD98059 alone or in combination with PI-103. For the NHEJ assay (Figure 6C), A549 cells stably expressing NHEJ repair platform were transiently transfected with a pDsRed-I-SceI expression vector with endonuclease activity. Twenty-four h after transfection, cells were treated with the inhibitor. Combined treatment significantly inhibited NHEJ activity (Figure 6C) and enhanced residual γ-H2AX foci in K-RASmut cells after 24 h treatment before IR exposure (Figure 6A–6B). Treatment with PD98059 alone for 2 h and/or 24 h or the combination of PD98059 and PI-103 treatment for 2 h did not inhibit NHEJ activity and the residual γ-H2AX foci (Figures 6A-6C).

Next, we tested whether the pattern of residual DSBs presented in Figure 5B correlates with post-irradiation cell survival. To this aim, confluent cells were treated with PI-103 (1 μM), PD98059 (20 μM) or the combination of PI-103 and PD98059 for 2 h or 24 h and irradiated with 4 Gy. The data obtained from survival of 4 Gy (SF4) indicated that PI-103 induces radiosensitization to the same degree when administered alone or in combination with PD98059 for 2 h. Following a 24 h pre-treatment, PI-103 alone did not induce radiosensitization, but the combination of PI-103 and PD98059 significantly reduced SF4 in both A549 and H460 cells (Figure 6D). Likewise, pretreatment with PD98059 for 2 h or 24 h did not increase radiation sensitivity in A549 and H460 cells (Figure 6D). Analyzing cell cycle progression revealed that treatment with PI-103 for 24 h does not attenuate the percentage of cells in radiosensitive G2 phase (data not shown). Thus, effect of cell cycle distribution on radiosensitization can be ruled out.

We also investigated the pattern of radiosensitization by PI-103 and PD98059 in K-RASwt NSCLC cell lines HTB-182 and SK-MES-1 after 2 h and 24 h pretreatment. Pretreatment with PI-103 alone or in combination with PD98059 for 2 h induced radiosensitization of HTB-182 cells. In contrast to the effect observed in K-RASmut cells, the pattern of radiosensitization by PI-103 in HTB-182 cells was not changed after 24 h pretreatment (Supplementary Figure S2). In SK-MES-1 cells, pretreatment with PI-103 for 2 h did not induce radiosensitization, most likely through slow rate of drug uptake in this cells. However, similar to the data obtained in K-RASwt HTB-182 cells, PI-103 induced radiosensitization after 24 h pretreatment. The level of radiosensitization remained unchanged after the combination of PI-103 and PD98059. Pretreatment with PD98059 induced radiosensitization in neither of the K-RASwt cells after 2 h pretreatment. After a 24 h ptretreatmt a slight radiosensitization was observed in HTB-182 cells only (Supplementary Figure S2).

Stimulation of radiation-induced apoptosis following the combination of molecular targeting strategies with ionizing radiation could be a mechanism that may lead to radiosensitization. This is also relevant to our study because Akt phosphorylation and inactivation of the pro-apoptotic proteins BAD and caspase 9 are able to diminish apoptosis induction after cellular stress [32]. Thus, we tested whether apoptosis is differentially induced in irradiated cells pre-treated with PI-103 and PD98059 alone or in combination. In our apoptosis FACS analysis, the fraction of apoptotic sub-G1 cells was significantly enhanced in H460 cells (P<0.001) but not in A549 cells 48 h after irradiation (Figure 6E). Interestingly, the combination of PI-103 and PD98059 with irradiation in 2 h or 24 h pretreatment slightly reduced the percentage of apoptotic cells. The combination of PI-103 and PD98059 in the un-irradiated condition markedly increased apoptosis in both cell lines, but this combination did not enhance the percentage of radiation-induced apoptosis (Figure 6E). The combination of PD98059 and irradiation led to significantly increased apoptosis only in A549 cells (P<0.01). It has been shown ZSTK474, a specific class I PI3K inhibitor leads to induction of autophagy in a concentration-dependent manner [33]. It is also known that therapeutic dose of ionizing radiation induces autophagy in cancer cells [34]. Therefore, we tested whether the combination of PI-103 and PD98059 with irradiation may lead to a change in autophagy induction compared to the either of treatments alone. Data shown in Supplementary Figure S3A indicates that the conversion of LC3-I to LC3-II as an autophagy marker is not markedly changed following 24 h treatment with ether of the inhibitor. However, the combination of PI-103 and PD98059 led to enhanced level of autophagy in un-irradiated as well as in 4 Gy irradiated condition 10 min post-IR in both cell lines (Supplementary Figure S3A).

To investigate whether combination of the inhibitors with irradiation changes the pattern of autophagy at longer time-point after irradiation, cells were treated with the inhibitors for 24 h as described in Supplementary Figure S3A and irradiated 4 Gy. Twenty-four h post-irradiation protein samples were isolated and levels of P-Akt (S473) and autophagy were analyzed. Data shown in Supplementary Figure S3B indicates neither PI-1103 nor PD98059 inhibited Akt phosphorylation analyzed 48 h after treatment. However, similar to the data shown in Figure 2B for 24 h treatment, the combined treatment with PI-103 and PD98059 for 48 h blocked phosphorylation of Akt under both un-irradiated and irradiated condition. No difference was observed regarding the conversion of LC3-II to LC3-I in irradiated and un-irradiated cells (Supplementary Figure S3B).

## DISCUSSION

We demonstrate here, for the first time, the MEK-ERK-dependent reactivation of Akt due to a 24 h inhibition of PI3K in K-RASmut NSCLC cell lines A549 and H460 *in vitro* and in A549 xenografts *in vivo*. Reactivation of Akt accelerated the repair of radiation-induced double-strand breaks (DSBs). Dual targeting of MEK and PI3K completely blocked Akt reactivation, impaired DSBs repair through NHEJ and improved radiosensitization.

Previously, we showed that targeting PI3K by PI-103 is a more effective strategy than targeting MEK to inhibit the clonogenic activity of tumor cells with constitutive K-Ras activity. Together with other reports [35, 36], this finding indicates that the survival of tumor cells expressing the K-Ras proto-oncogene mainly depends on the PI3K/Akt pathway. In general and as shown especially in xenografts of K-RASmut colorectal carcinomas, disease stabilization by MEK and PI3K inhibition might not translate into a durable therapeutic effect [37]. Thus, a combination of molecular targeting strategies with alternative curative cancer therapy approaches such as radiotherapy is necessary in K-RASmut cancers.

DSBs are the prime cause of cell-death induced by radiotherapy. Several reports indicate that the repair of ionizing radiation-induced DSBs and resistance to radiotherapy partially depends on the activation of the PI3K/Akt pathway *in vitro* and *in vivo* [23, 24, 26, 38, 39]. With regard to the specific role of Akt in DSBs repair, we were the first to demonstrate that Akt1 physically binds to DNA-PKcs and induces its activity, which is essential for the initiation, progression, and termination of the NHEJ repair pathway [20–22]. Thus, according to the previously described role of Akt in DSBs repair [20–22], and as shown in the present study, complete inhibition of Akt during irradiation leads to impaired repair of DSBs and results in radiosensitization. Likewise, the described MEK/ERK-dependent reactivation of Akt following 24 h pretreatment with PI3K inhibitors accelerates repair of DSBs and results in a lack of radiosensitization (Figure 6), independent of the regulation of radiation-induced apoptosis or changes in autophagy induction. It is important to note that the initial number of radiation-induced DSBs and consequently the frequency of γH2AX foci is similar in cells with and without functional Akt [21].

In the A549 xenograft pretreatment with PI-103, the inhibition of phosphorylation of Akt and DNA-PKcs at 30 min post-irradiation is in line with the Akt-dependent activation of DNA-PKcs [21–23]. In contrast, in those tumors that were treated with PI-103, Akt activity was markedly enhanced, while DNA-PKcs phosphorylation was significantly inhibited. According to the report by Raynaud *et al.* [40], this effect of PI-103 on DNA-PKcs phosphorylation might be due to an Akt-independent direct inhibition of DNA-PKcs [40]. Although direct inhibition of DNA-PKcs phosphorylation in K-RASwt H661 cells could not be shown, nevertheless according to Raynaud et al. [40], we propose two modes of action of PI-103 on DNA-PKcs phosphorylation after irradiation; 1) PI-103 inhibits DNA-PKcs phosphorylation through a direct inhibition of DNA-PKcs, and 2) because DNA-PKcs is a substrate of Akt [21, 22, 41], PI-103 reduces DNA-PKcs activity indirectly through inhibition of Akt. In this study, pretreatment with PI-103 for 24 h led to the significant reactivation of Akt followed by enhanced radiation-induced DNA-PKcs activity, leading to the lack of inhibition of DNA-PKcs by PI-103. Thus, the final effect of PI-103 on DNA-PKcs depends on the intensity of the pathways and the interplay between the two different signals. Data from A549 xenograft tumors demonstrates that the reactivation of Akt after 24 h pretreatment with PI-103 is a dominant signal. This signal is strong enough to mask the direct inhibitory effect of PI-103 on DNA-PKcs. This leads to the lack of a final effect on the DNA-PKcs activity. In contrast, as shown in Figure 4B and the densitometry analyses in Figure 4F, reactivation of activated Akt at the 3 h pretreatment time point is still not strong. Therefore, significant inhibition of DNA-PKcs phosphorylation by the PI3K inhibitor was observed. In the *in vivo* study, it was shown that Akt activity at one hour after treatment (group A) is markedly inhibited by PI-103 (Figure 4). At 3.5 h post-treatment (group B), phospho-Akt is not inhibited by PI-103. Even the densitometry analysis indicates a slight but not significant reactivation of Akt by PI3K inhibition at this time point. According to these data, it seems that Akt reactivation by PI-103 appears in the *in vivo* conditions at a significantly shorter time than in the *in vitro* conditions, which usually starts approximately 16 h post-treatment (Figure 5A). Thus, the effect of Akt reactivation on cell survival especially after irradiation *in vivo* might be more important than the effect *in vitro*.

Based on this mechanistic study, single targeting of PI3K in a K-RASmut tumor *in vivo* does not seem to be an effective approach to overcome radioresistance. Thus, combination treatment with PI3K and radiotherapy may not improve treatment outcome. The alternative approach will be co-targeting of PI3K and MEK combined with standard radiotherapy as shown in the present study *in vitro*. The effectiveness of the combination of PI3K and MEK inhibitors with radiotherapy in our study is in line with the data obtained by Williams *et al.* [42] using pancreatic cancer cells treated with a similar strategy. In this context it is important to know that 90% of cancers present a *K-RAS* mutation [43]. Williams *et al.* [42], however, did not investigate the crosstalk between the two pathways and the mechanism of action of the combination of PI3K and MEK inhibitors with radiotherapy [42].

**Figure 7:**
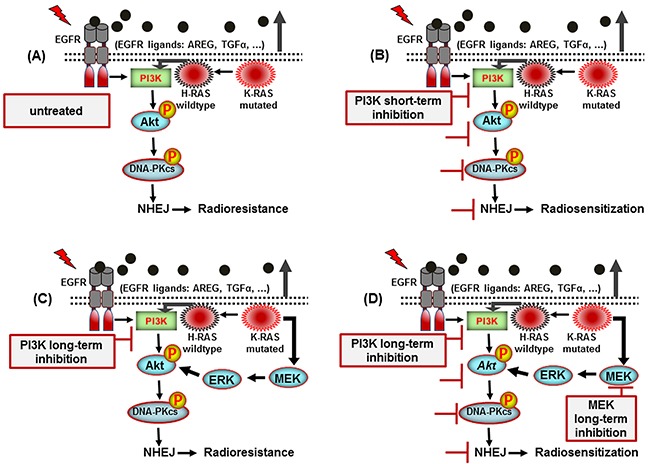
A model illustrating the signaling pathways involved in post-irradiation cells survival of K-RAS mutated NSCLC cells **A.** K-RAS mutation activity enhances NHEJ repair through Akt leading to radioresistance. **B.** Short-term inhibition of PI3K leads to inhibition of DNA-PKcs and radiosensitization. **C.** While long-term treatment reactivates Akt which mediates radioresistance. **D.** Long-term dual-targeting of PI3K and MEK is an efficient approch to block Akt reactivation and improve radiotherapy outcome.

As summarized in Figure 7, our study revealed that short-term treatment with PI-103 inhibits Akt reactivation and DNA-PKcs phosphorylation following radiotherapy in K-RASmut NSCLC tumors. We also demonstrate a mechanism that impairs the efficacy of PI3K targeting to improve the response of K-RASmut tumor cells to radiotherapy. Thus, to our knowledge, this is the first study to exhibit that dual targeting of PI3K and MEK provides an effective strategy to overcome the therapeutic resistance of currently available PI3K inhibitors combined with irradiation in K-RASmut cancers.

## MATERIALS AND METHODS

### Antibodies and reagents

Antibodies against phospho-Akt-S473 (Cat. # 9271), phospho-Akt-T308, (Cat. # 9275), phospho-PRAS40-T246 (Cat. # 2997), PRAS40 (Cat. # 2691), phospho-ERK1/2-T202/Y204, (Cat. # 4377), ERK1/2 (Cat. # 4695), P-ATM (S1981) (Cat. # 5883), ATM (Cat. # 2873), phospho-ATR-S428 (Cat. # 2585), ATR (Cat. # 2790) phosphospho-Chk1-S345 (Cat. # 2341), Chk1 (Cat. # 2360), phospho-Chk2-T68 (Cat. # 2197), Chk2 (Cat. # 6334), phospho-mTOR-S2448 (Cat. # 2971), phospho-mTOR-S2481 (Cat. # 2974) and mTOR (Cat. # 2983) were purchased from Cell Signaling (Frankfurt, Germany). Phospho-DNA-PKcs-S2056 (ab18192) and DNA-PKcs (Cat. # ab1832) antibodies were purchased from Abcam (Cambridge, UK). PI-103 (Cat. # P-9099) was purchased from LC laboratories (Woburn, MA, USA). The PI3K inhibitor LY294002 (Cat. # 440202) was purchased from Merck KGaA (Darmstadt, Germany). Anti-phospho-Histone H2AX (Ser139) (Cat. # 05-636) antibody was purchased from Merck Millipore (Darmstadt, Germany). LC3 antibody (Cat # 5F10) was purchased from nanoTools (Teningen, Germany).

### Cell lines

The established K-RASmut NSCLC cell lines; A549 (ATCC, CCL-185™), H460 (ATCC, HTB-177™) and K-RASwt NSCLC cell lines H661 (ATCC, HTB-183D™), HTB-182 and SK-MES-1 were used. An authentication test for A549 and H460 cells was performed by Multiplexion (Immenstaad, Germany). Cells were cultured in DMEM (A549, HTB-182 and SK-MES-1) or RPMI-1640 (H460 and H661) (Gibco) routinely supplemented with 10% FCS and 1% penicillin-streptomycin and incubated in a humidified atmosphere with 93% air/7% CO_2_ at 37°C. Cells were regularly tested for mycoplasma contamination. A549 cells stably transfected with NHEJ reporter constructs were kindly provided by Dr. Malte Kriegs (Laboratory of Radiobiology and Experimental Radiooncology, University Medical Center Hamburg-Eppendorf, Hamburg, Germany).

### Flow cytometric analysis of apoptosis and clonogenic assay

Cells were seeded in 60 mm culture dishes (75×10^3^/dish) and 24 h later were treated with DMSO, PI-103 (1 μM), PD98059 (20 μM) or the combination of PI-103 and PD98059. The final concentration of DMSO in all conditions was adjusted to be similar (0.1%). At 2 h or 24 h post-treatment, cultures were mock irradiated or irradiated with 4 Gy. Forty-eight hours after irradiation, cells were prepared for FACS as described earlier [21]. To investigate the effect of the PI3K inhibitor PI-103 alone or in combination with the MEK inhibitor PD98059 on post-irradiation cell survival, a clonogenic assay was performed. To this end, confluent cells were treated with DMSO, PI-103 (1 μM), PD98059 (20 μM) or a combination PI-103 and PD98059 for 2 h or 24 h. Thereafter, cells were irradiated with 4 Gy and plated in 6-well plates 2 h post-irradiation. Ten to 12 days later, the culture dishes were stained with 0.05% w/v crystal violet solution, and the clonogenic fraction of irradiated cells was calculated [20].

### SiRNA transfection, protein extraction and western blotting

Cells were transiently transfected with 50 nM siRNA against K-RAS (Dharmacon, #M-005069), MEK1 (Cell Signaling, #6423), ERK2 (Darmacon, #M-003555) or non-targeting siRNA (Dharmacon, #D-001810) using lipofectamine or lipofectamine RNAiMAX according to the manufacturer's instructions (Life Technologies, Darmstadt, Germany). Immunoblot analysis was conducted with the transfected cells three days after transfection. To analyze protein expression and activity after the indicated treatments in each experiment, cells were washed twice with PBS and then lysed with lysis buffer [30]. Immunoblotting was performed as previously described [4].

### Non-homologous end-joining repair assay

A NHEJ repair assay was performed in A549 cells stably transfected with NHEJ reporter constructs as described previously [44, 45]. In brief, cells were transiently transfected with 1 μg of the p-I-SceI expression vector by electroporation using an Amaxa nucleofector II device. Twenty-four h after transfection, confluent cells were treated with inhibitors. After an additional 24 h, cells were harvested, and the percentage of GFP positive cells was determined using a flow cytometer.

### γH2AX foci assay and confocal microscopy

A γH2AX foci assay was used to determine the amount of residual DSBs as described earlier [20]. In brief, confluent cells grown on chamber slides were treated with inhibitors as described for the clonogenic assay. Cells were irradiated with 4 Gy and were fixed after 24 h and permeabilized by 0.2% Triton X-100. Thereafter, samples were blocked with PBS/3% BSA and incubated with anti-phospho-histone H2AX antibody for 1 hour followed by washing and incubation with second antibody. After washing, slides were mounted using VECTASHIELD Mounting Medium with 4′, 6-diamidino-2-phenylindole (Vector Laboratories, Peterborough, UK). The slides were viewed at 400x magnification on a fluorescence microscope (Axioplan 2, Zeiss); residual γ-H2AX foci were counted and graphed.

To perform confocal microscopy, cultured cells on culture slides were irradiated with 4 Gy following 2 h pre-treatment with PI-103 and fixed with periodate-lysine-paraformaldehyde 24 h after irradiation. For immunofluorescence analysis, cells were incubated with mouse anti-P-H2AX (S139) and rabbit anti-phospho-DNA-PKcs (S2056) antibodies at room temperature for one hour. Bound antibodies were visualized by incubation with a Cy3-donkey anti-mouse and Cy5-donkey anti-rabbit antibodies for 30 min. Nuclei were stained with YO-PRO and analyzed with a confocal laser scanning microscope (Leica TCS SP, Leica Microsystems, Bensheim, Germany).

### Mouse human tumor xenografts

Athymic nude mice (4- to 6-week-old males) were obtained from Harlan Laboratories. All animal procedures and maintenance were conducted in accordance with the institutional guidelines of the University of Wisconsin. Mice were injected with A549 (2 × 10^6^ cells) in both hind legs, and tumors were allowed to grow to approximately 250 mm^3^. Mice were randomized and gavaged with either 5 mg/kg of PI-103 or vehicle (1% DMSO + 30% polyethylene glycol + 1% tween 80 in PBS). Tumors were locally irradiated with an XRAD320 (Precision X-RAY, Branford, CT, USA), with a single dose of 4 Gy at 30 min, 3 h or 24 h post-treatment with PI-103. Mice pretreated with PI-103 for 30 min (group A) and 3 h (group B) were euthanized 30 min after irradiation, while those mice pretreated with PI-103 for 24 h 3 h (group C) were euthanized 24 h after irradiation. Tumors were harvested, and protein samples were isolated for Western blotting.

### Statistics and densitometry

Student's *t*-test was used to compare the data between two groups. *P* < 0.05 was considered statistically significant (*P < 0.05; **P < 0.01; ***P < 001). Densitometric quantifications of the immunoblots were performed with *ImageJ* software (http://rsbweb.nih.gov/ij/).

## SUPPLEMENTARY FIGURES



## References

[1] Maertens O, Cichowski K (2014). An expanding role for RAS GTPase activating proteins (RAS GAPs) in cancer. Adv Biol Regul.

[2] Toulany M, Baumann M, Rodemann HP (2007). Stimulated PI3K-AKT signaling mediated through ligand or radiation-induced EGFR depends indirectly, but not directly, on constitutive K-Ras activity. Mol Cancer Res.

[3] Linardou H, Dahabreh IJ, Kanaloupiti D, Siannis F, Bafaloukos D, Kosmidis P, Papadimitriou CA, Murray S (2008). Assessment of somatic k-RAS mutations as a mechanism associated with resistance to EGFR-targeted agents: a systematic review and meta-analysis of studies in advanced non-small-cell lung cancer and metastatic colorectal cancer. Lancet Oncol.

[4] Toulany M, Schickfluss TA, Eicheler W, Kehlbach R, Schittek B, Rodemann HP (2011). Impact of oncogenic K-RAS on YB-1 phosphorylation induced by ionizing radiation. Breast Cancer Res.

[5] Affolter A, Drigotas M, Fruth K, Schmidtmann I, Brochhausen C, Mann WJ, Brieger J (2013). Increased radioresistance via G12S K-Ras by compensatory upregulation of MAPK and PI3K pathways in epithelial cancer. Head Neck.

[6] Saki M, Toulany M, Rodemann HP (2013). Acquired resistance to cetuximab is associated with the overexpression of Ras family members and the loss of radiosensitization in head and neck cancer cells. Radiother Oncol.

[7] Bernhard EJ, Stanbridge EJ, Gupta S, Gupta AK, Soto D, Bakanauskas VJ, Cerniglia GJ, Muschel RJ, McKenna WG (2000). Direct evidence for the contribution of activated N-ras and K-ras oncogenes to increased intrinsic radiation resistance in human tumor cell lines. Cancer Res.

[8] Wegman P, Ahlin C, Sorbe B (2011). Genetic alterations in the K-Ras gene influence the prognosis in patients with cervical cancer treated by radiotherapy. Int J Gynecol Cancer.

[9] Minjgee M, Toulany M, Kehlbach R, Giehl K, Rodemann HP (2011). K-RAS(V12) induces autocrine production of EGFR ligands and mediates radioresistance through EGFR-dependent Akt signaling and activation of DNA-PKcs. Int J Radiat Oncol Biol Phys.

[10] Toulany M, Dittmann K, Kruger M, Baumann M, Rodemann HP (2005). Radioresistance of K-Ras mutated human tumor cells is mediated through EGFR-dependent activation of PI3K-AKT pathway. Radiother Oncol.

[11] Gupta S, Ramjaun AR, Haiko P, Wang Y, Warne PH, Nicke B, Nye E, Stamp G, Alitalo K, Downward J (2007). Binding of ras to phosphoinositide 3-kinase p110alpha is required for ras-driven tumorigenesis in mice. Cell.

[12] Cengel KA, Voong KR, Chandrasekaran S, Maggiorella L, Brunner TB, Stanbridge E, Kao GD, McKenna WG, Bernhard EJ (2007). Oncogenic K-Ras signals through epidermal growth factor receptor and wild-type H-Ras to promote radiation survival in pancreatic and colorectal carcinoma cells. Neoplasia.

[13] Ciuffreda L, Di Sanza C, Cesta Incani U, Eramo A, Desideri M, Biagioni F, Passeri D, Falcone I, Sette G, Bergamo P, Anichini A, Sabapathy K, McCubrey JA (2012). The mitogen-activated protein kinase (MAPK) cascade controls phosphatase and tensin homolog (PTEN) expression through multiple mechanisms. J Mol Med (Berl).

[14] Zmajkovicova K, Jesenberger V, Catalanotti F, Baumgartner C, Reyes G, Baccarini M (2013). MEK1 is required for PTEN membrane recruitment, AKT regulation, and the maintenance of peripheral tolerance. Mol Cell.

[15] Will M, Qin AC, Toy W, Yao Z, Rodrik-Outmezguine V, Schneider C, Huang X, Monian P, Jiang X, de Stanchina E, Baselga J, Liu N, Chandarlapaty S, Rosen N (2014). Rapid induction of apoptosis by PI3K inhibitors is dependent upon their transient inhibition of RAS-ERK signaling. Cancer Discov.

[16] Toulany M, Minjgee M, Saki M, Holler M, Meier F, Eicheler W, Rodemann HP (2014). ERK2-dependent reactivation of Akt mediates the limited response of tumor cells with constitutive K-RAS activity to PI3K inhibition. Cancer Biol Ther.

[17] Jackson SP (2002). Sensing and repairing DNA double-strand breaks. Carcinogenesis.

[18] Chang L, Graham PH, Hao J, Ni J, Bucci J, Cozzi PJ, Kearsley JH, Li Y (2014). PI3K/Akt/mTOR pathway inhibitors enhance radiosensitivity in radioresistant prostate cancer cells through inducing apoptosis, reducing autophagy, suppressing NHEJ and HR repair pathways. Cell Death Dis.

[19] Liu WL, Gao M, Tzen KY, Tsai CL, Hsu FM, Cheng AL, Cheng JC (2014). Targeting Phosphatidylinositide3-Kinase/Akt pathway by BKM120 for radiosensitization in hepatocellular carcinoma. Oncotarget.

[20] Toulany M, Kasten-Pisula U, Brammer I, Wang S, Chen J, Dittmann K, Baumann M, Dikomey E, Rodemann HP (2006). Blockage of epidermal growth factor receptor-phosphatidylinositol 3-kinase-AKT signaling increases radiosensitivity of K-RAS mutated human tumor cells in vitro by affecting DNA repair. Clin Cancer Res.

[21] Toulany M, Kehlbach R, Florczak U, Sak A, Wang S, Chen J, Lobrich M, Rodemann HP (2008). Targeting of AKT1 enhances radiation toxicity of human tumor cells by inhibiting DNA-PKcs-dependent DNA double-strand break repair. Mol Cancer Ther.

[22] Toulany M, Lee KJ, Fattah KR, Lin YF, Fehrenbacher B, Schaller M, Chen BP, Chen DJ, Rodemann HP (2012). Akt promotes post-irradiation survival of human tumor cells through initiation, progression, and termination of DNA-PKcs-dependent DNA double-strand break repair. Mol Cancer Res.

[23] Choi EJ, Ryu YK, Kim SY, Wu HG, Kim JS, Kim IH, Kim IA (2010). Targeting epidermal growth factor receptor-associated signaling pathways in non-small cell lung cancer cells: implication in radiation response. Mol Cancer Res.

[24] Fraser M, Harding SM, Zhao H, Coackley C, Durocher D, Bristow RG (2011). MRE11 promotes AKT phosphorylation in direct response to DNA double-strand breaks. Cell cycle.

[25] No M, Choi EJ, Kim IA (2009). Targeting HER2 signaling pathway for radiosensitization: alternative strategy for therapeutic resistance. Cancer Biol Ther.

[26] Kao GD, Jiang Z, Fernandes AM, Gupta AK, Maity A (2007). Inhibition of phosphatidylinositol-3-OH kinase/Akt signaling impairs DNA repair in glioblastoma cells following ionizing radiation. J Biol Chem.

[27] Meyn RE, Munshi A, Haymach JV, Milas L, Ang KK (2009). Receptor signaling as a regulatory mechanism of DNA repair. Radiother Oncol.

[28] O'Reilly KE, Rojo F, She QB, Solit D, Mills GB, Smith D, Lane H, Hofmann F, Hicklin DJ, Ludwig DL, Baselga J, Rosen N (2006). mTOR inhibition induces upstream receptor tyrosine kinase signaling and activates Akt. Cancer Res.

[29] Rodrik-Outmezguine VS, Chandarlapaty S, Pagano NC, Poulikakos PI, Scaltriti M, Moskatel E, Baselga J, Guichard S, Rosen N (2011). mTOR kinase inhibition causes feedback-dependent biphasic regulation of AKT signaling. Cancer Discov.

[30] Toulany M, Dittmann K, Baumann M, Rodemann HP (2005). Radiosensitization of Ras-mutated human tumor cells in vitro by the specific EGF receptor antagonist BIBX1382BS. Radiother Oncol.

[31] Uematsu N, Weterings E, Yano K, Morotomi-Yano K, Jakob B, Taucher-Scholz G, Mari PO, van Gent DC, Chen BP, Chen DJ (2007). Autophosphorylation of DNA-PKCS regulates its dynamics at DNA double-strand breaks. J Cell Biol.

[32] Nicholson KM, Anderson NG (2002). The protein kinase B/Akt signalling pathway in human malignancy. Cell Signal.

[33] Wang Y, Liu J, Qiu Y, Jin M, Chen X, Fan G, Wang R, Kong D (2016). ZSTK474, a specific class I phosphatidylinositol 3-kinase inhibitor, induces G1 arrest and autophagy in human breast cancer MCF-7 cells. Oncotarget.

[34] Chaachouay H, Ohneseit P, Toulany M, Kehlbach R, Multhoff G, Rodemann HP (2011). Autophagy contributes to resistance of tumor cells to ionizing radiation. Radiother Oncol.

[35] Falasca M, Selvaggi F, Buus R, Sulpizio S, Edling CE (2011). Targeting phosphoinositide 3-kinase pathways in pancreatic cancer--from molecular signalling to clinical trials. Anticancer Agents Med Chem.

[36] Hubbard PA, Moody CL, Murali R (2014). Allosteric modulation of Ras and the PI3K/AKT/mTOR pathway: emerging therapeutic opportunities. Front Physiol.

[37] Migliardi G, Sassi F, Torti D, Galimi F, Zanella ER, Buscarino M, Ribero D, Muratore A, Massucco P, Pisacane A, Risio M, Capussotti L, Marsoni S (2012). Inhibition of MEK and PI3K/mTOR suppresses tumor growth but does not cause tumor regression in patient-derived xenografts of RAS-mutant colorectal carcinomas. Clin Cancer Res.

[38] Gupta AK, McKenna WG, Weber CN, Feldman MD, Goldsmith JD, Mick R, Machtay M, Rosenthal DI, Bakanauskas VJ, Cerniglia GJ, Bernhard EJ, Weber RS, Muschel RJ (2002). Local recurrence in head and neck cancer: relationship to radiation resistance and signal transduction. Clin Cancer Res.

[39] Kim TJ, Lee JW, Song SY, Choi JJ, Choi CH, Kim BG, Lee JH, Bae DS (2006). Increased expression of pAKT is associated with radiation resistance in cervical cancer. Br J Cancer.

[40] Raynaud FI, Eccles S, Clarke PA, Hayes A, Nutley B, Alix S, Henley A, Di-Stefano F, Ahmad Z, Guillard S, Bjerke LM, Kelland L, Valenti M (2007). Pharmacologic characterization of a potent inhibitor of class I phosphatidylinositide 3-kinases. Cancer Res.

[41] Westhoff MA, Kandenwein JA, Karl S, Vellanki SH, Braun V, Eramo A, Antoniadis G, Debatin KM, Fulda S (2009). The pyridinylfuranopyrimidine inhibitor, PI-103, chemosensitizes glioblastoma cells for apoptosis by inhibiting DNA repair. Oncogene.

[42] Williams TM, Flecha AR, Keller P, Ram A, Karnak D, Galban S, Galban CJ, Ross BD, Lawrence TS, Rehemtulla A, Sebolt-Leopold J (2012). Cotargeting MAPK and PI3K signaling with concurrent radiotherapy as a strategy for the treatment of pancreatic cancer. Mol Cancer Ther.

[43] Almoguera C, Shibata D, Forrester K, Martin J, Arnheim N, Perucho M (1988). Most human carcinomas of the exocrine pancreas contain mutant c-K-ras genes. Cell.

[44] Kriegs M, Kasten-Pisula U, Rieckmann T, Holst K, Saker J, Dahm-Daphi J, Dikomey E (2010). The epidermal growth factor receptor modulates DNA double-strand break repair by regulating non-homologous end-joining. DNA Repair (Amst).

[45] Myllynen L, Rieckmann T, Dahm-Daphi J, Kasten-Pisula U, Petersen C, Dikomey E, Kriegs M (2011). In tumor cells regulation of DNA double strand break repair through EGF receptor involves both NHEJ and HR and is independent of p53 and K-Ras status. Radiother Oncol.

