# Supplementary material for: Dual targeting of PI3K and MEK enhances the radiation response of K-RAS mutated non-small cell lung cancer
Source: Oncotarget. 2016 May 27;7(28):43746–61. doi: 10.18632/oncotarget.9670 (PMC5190057; doi:10.18632/oncotarget.9670)
Supplement: Supplementary file 1 [file oncotarget-07-43746-s001.pdf]

SUPPLEMENTARY FIGURES

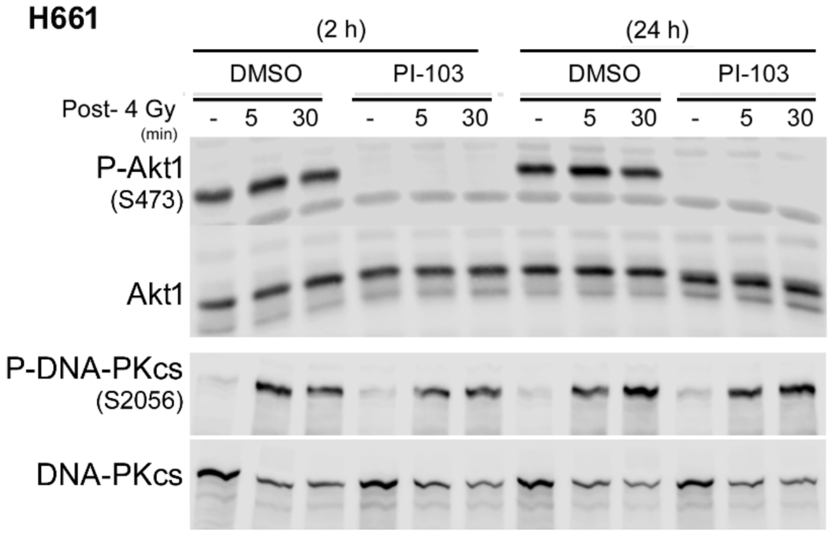

Supplementary Figure S1: Effect of PI-103 on Akt and DNA-PKcs phosphorylation after irradiation in K-RASwt H661 cells. Cells were treated with PI-103 for 2 h or 24 h and mock irradiated or irradiated with 4 Gy. Protein samples were isolated at the indicated time-points after irradiation and subjected to SDS-PAGE. The levels of P-DNA-PKcs (S2056), P-Akt (S473), were analyzed by Western blotting. Blots were stripped and incubated with antibodies against DNA-PKcs and Akt1.

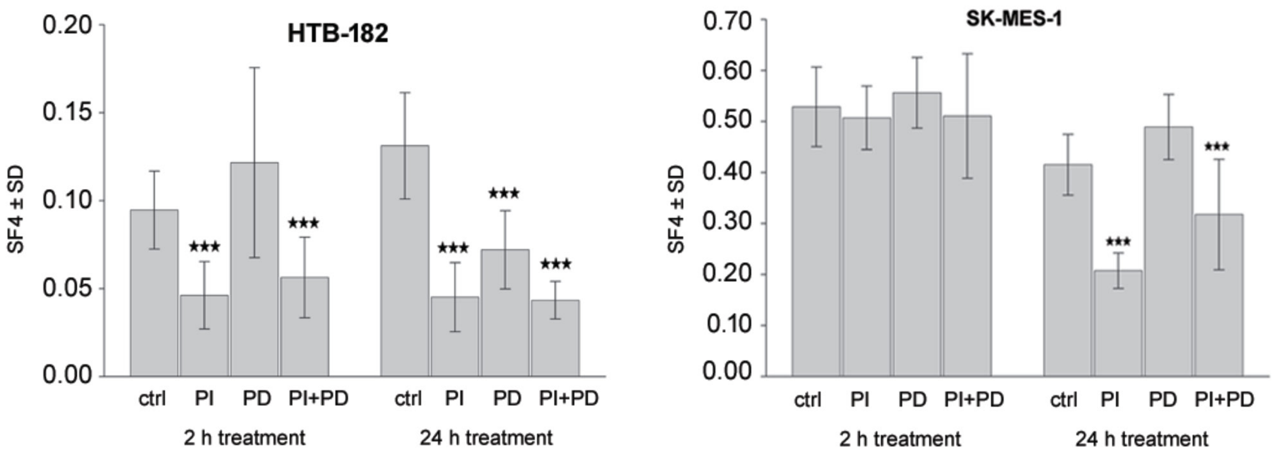

Supplementary Figure S2: Effect of long-term treatment with PI-103 alone or in combination with PD98059 induces radiosensitization of K-RASwt NSCLC cells. Survival fraction after 4 Gy (SF4) from clonogenic assay performed 2 h post-irradiation. The SF4 value is the mean survival fraction obtained from 24 data points from two independent experiments (****P*<0.001, Student's *t*-test); (ctrl: control, PI: PI-103, PD: PD98059); bars: SD.

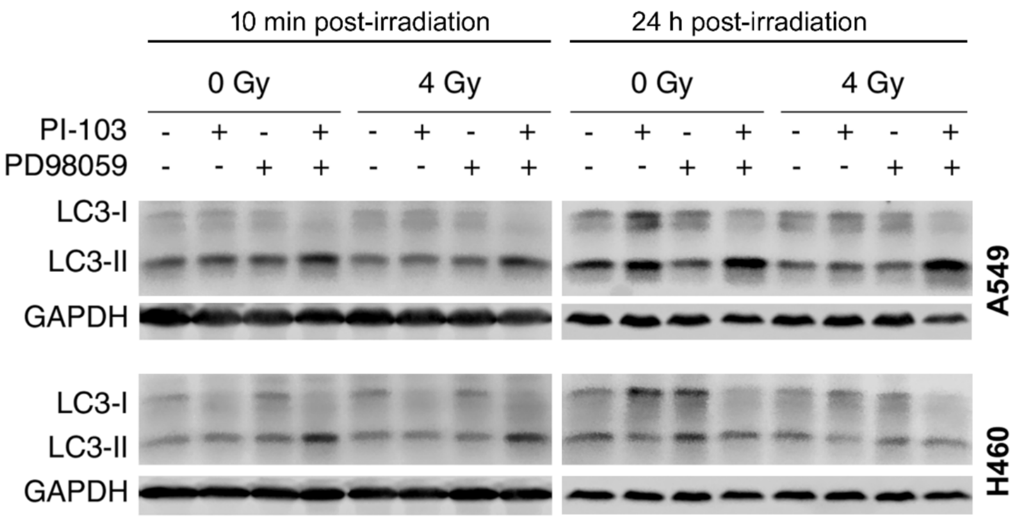

Supplementary Figure S3: Autophagy induction by pretreatment with PI-103, PD98059 and the combination of PI-103 and PD98059 for 24 h followed by mock irradiation or irradiation with 4 Gy. Cells were treated with the inhibitor for 24 h and mock irradiated or irradiated with 4 Gy. Protein samples were isolated at 10 min post-irradiation and subjected to SDS-PAGE. Level of LC3 was analyzed Western blotting. GAPDH was detected as loading control.
